# Validated metabolomic biomarkers in psychiatric disorders: a narrative review

**DOI:** 10.1186/s10020-025-01258-7

**Published:** 2025-07-09

**Authors:** Marcela Konjevod, Jorge Sáiz, Lucía Bordoy, Dubravka Svob Strac, Ameer Y. Taha, Senentxu Lanceros-Méndez, Rosa M. Alonso

**Affiliations:** 1 Laboratory for Molecular Neuropsychiatry, Division of Molecular Medicine, Ruder Boskovic Institute, 10000 Zagreb, Croatia; 2 FARMARTEM Group, Department of Analytical Chemistry, Faculty of Science and Technology, University of the Basque Country (UPV/EHU), 48940 Leioa, Biscay, Spain; 3 Department of Food Science and Technology, College of Agriculture and Environmental Sciences, University of California, Davis, CA USA; 4 BCMaterials, Basque Center for Materials, Applications and Nanostructures, UPV/EHU Science Park, 48940 Leioa, Spain; 5 Dr. Rodríguez Lafora Hospital, Madrid, Spain; 6 Ikerbasque, Basque Foundation for Science, 48009 Bilbao, Spain

**Keywords:** Validation, Biomarkers, Metabolomics, Psychiatric disorders

## Abstract

Schizophrenia, major depressive disorder, bipolar disorder and posttraumatic stress disorder are severe and profoundly debilitating mental illnesses. Due to their heterogeneity and polygenic nature, the metabolic pathways and biological mechanisms underlying these conditions remain elusive. Consequently, diagnosing psychiatric disorders is a complex and multifaceted process, relying on clinical assessment and standardized diagnostic criteria. There is a growing demand to identify and integrate potential biomarkers for these disorders, especially for early diagnosis, to enhance diagnostic accuracy and complement existing diagnostic tools. Validating potential diagnostic biomarkers is essential to ensure their accuracy, reliability, generalizability, and clinical utility.

In this article we provide a comprehensive review of validated metabolomics research, focusing on both the specific psychiatric conditions and the types of validation performed. Our scope is limited to peer-reviewed studies that include studies that performed validation studies in independent cohorts, cross-validation, or external validation. Due to the lack of published research, most of these validation studies have concentrated on major depressive disorder and schizophrenia, with fewer studies addressing bipolar disorder and posttraumatic stress disorder.

Biomarkers are considered as validated if they demonstrated reproducibility in additional cohorts and biological relevance across independent datasets. However, several limitations must be acknowledged, including the heterogeneity in study design, small sample sizes, different analytical platforms, and inconsistent validation criteria across studies. Published results reveal that potential metabolomics biomarkers pertain to diverse categories pointing to a range of cellular, biological, and metabolic processes and emphasizing the intricate nature of psychiatric disorders. Such findings illustrate the formidable challenge of identifying and validating clinically useful metabolomic biomarkers and underscore the necessity for further research that encompasses various validation methodologies.

## Introduction

**Fig. 1:**
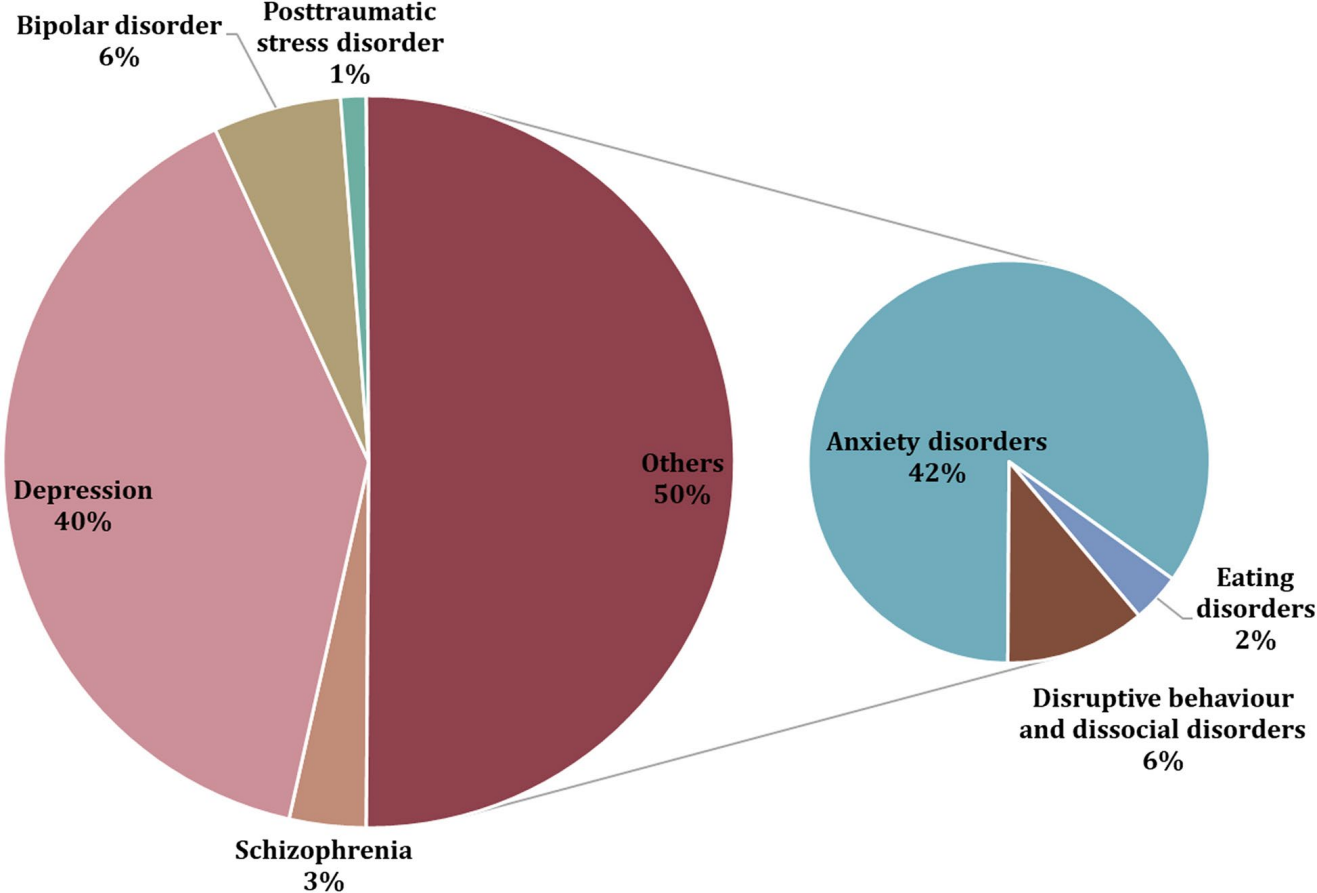
The most prevalent psychiatric disorders worldwide according to World Health Organization (WHO) (World Health Organization 2022)

Psychiatric disorders, encompassing a variety of prevalent brain conditions, such as schizophrenia (SCZ), major depressive disorder (MDD), bipolar disorder (BD), and posttraumatic stress disorder (PTSD), are severe and debilitating mental disorders (Zuo et al. 2021; Nedic Erjavec et al. 2018). These illnesses are multisystem disorders characterized by a spectrum of various symptoms and disturbances affecting psychomotor, emotional and socio-cognitive functioning (Zuo et al. 2021; Glannon 2022). Affecting an estimated 800 million individuals worldwide (Fig. 1), these conditions represent a significant public health challenge, with their etiology remaining elusive due to the intricate interplay of genetic, biological, and environmental factors (Zuo et al. 2021; Nedic Erjavec et al. 2018). Owing to the heterogeneity and polygenic nature of these conditions, the metabolic pathways and biological mechanisms underpinning their development and progression are still not well understood (Nedic Erjavec et al. 2018; Sethi and Brietzke 2015; Hayashi-Takagi et al. 2014). Additionally, there is a high rate of comorbidity and overlap in neural dysfunction among psychiatric disorders, as they arise from diverse and complex psychopathological factors (Sethi and Brietzke 2015). Therefore, the diagnosis of psychiatric disorders represents a complex and multifaceted process that relies on clinical assessment (clinical interviews and patient history), standardized diagnostic criteria (Diagnostic and Statistical Manual of Mental Disorders, Fifth Edition – DSM-V, International Classification of Diseases, Eleventh Revision – ICD-11) and often, the use of various assessment tools (questionnaires, neuroimaging, laboratory tests) (American Psychiatric Association 2013; Almeida et al. 2020; Kay et al. 1987). These diagnostic approaches facilitate the standardization, comprehension, and classification of symptoms, as well as the definition of researched populations and therapeutic interventions. Nevertheless, the diagnosis of psychiatric disorders is fraught with several limitations. It predominantly hinges on clinical judgment, which can be subjective and vary between clinicians. Additionally, many psychiatric disorders have overlapping symptoms, which together with the lack of clear biological markers is making differential diagnosis challenging (Kendler and First 2010; Kupfer and Regier 2011; Kupfer et al. 2013; Brady et al. 2000; Fuchs 2010).

Therefore, there is an increasing demand for the identification and determination of biomarkers, in particular for early diagnosis of psychiatric disorders (Lozupone et al. 2019). By identifying molecular mechanisms and metabolic pathways involved in their pathogenesis, it would be possible to enhance early and accurate diagnosis, predict disease trajectory, and monitor treatment responses. Advanced high-throughput techniques such as genomics, transcriptomics, proteomics, metabolomics, or other omics approaches, could enlighten the complex mechanisms underlying the development and progression of psychiatric disorders (Sethi and Brietzke 2015). However, psychiatric conditions are multisystem disorders with great variability, causing dysregulations not only in the brain, but in the whole organism, as well (Glannon 2022). Therefore, the potential biomarkers for psychiatric conditions would likely consist of clusters of metabolites, resulting from the various affected metabolic pathways.

Variability of metabolite levels can be influenced by numerous confounding factors such as comorbid medical conditions, lifestyle factors (e.g., diet, sleep, stress), and the use of pharmacological treatments. Many psychiatric patients are on polypharmacy regimens, and medications, which can significantly alter metabolic profiles, Likewise, one of the foremost challenges in the metabolomic studies for biomarker discovery and identification is the integration of the vast data sets, generated by different analytical techniques in different research centers and laboratories employing different analytical methodologies (Izumi et al. 2019). Such integration is often limited due to various factors regarding the experimental design, data treatment and statistical methods, as well as lack of standardized protocols across metabolomic studies (Pinu et al. 2019; Li et al. 2024). Differences in sample collection, storage conditions, analytical platforms, data processing, and statistical analysis create inconsistencies that hinder reproducibility and cross-study comparisons. These differences further complicate the assurance of reliable and reproducible metabolomic metabolites (Izumi et al. 2019; Pinu et al. 2019; Li et al. 2024; Ulmer et al. 2021). Hence, to ensure their specificity, reliability, speed, and consistency, potential metabolomic biomarkers must undergo rigorous validation prior introduction into clinical practice (Izumi et al. 2019).

Moreover, the current literature often lacks rigorous validation steps, with many studies identifying differential metabolites without subsequent replication or confirmation in larger, independent samples. This contributes to a fragmented and sometimes contradictory results. As a result, although metabolomic studies provide valuable insights into dysregulated biochemical pathways, their clinical applicability remains limited without standardized methodologies and comprehensive validation analyses.

## Diagnosis of psychiatric disorders

Current diagnostic tools, including structured interviews and standardized questionnaires, are of the main importance in the diagnosis of psychiatric disorders. However, they rely heavily on self-reported symptoms and clinician observation (Kapur et al. 2012). In this section, we discuss the specific characteristics of SCZ, MDD, BD, and PTSD and their diagnostic methods in clinical praxis.

### Schizophrenia

SCZ is serious mental health condition categorized within the group of psychotic disorders, which are characterized by a loss of contact with reality and impaired cognitive functioning. The onset of SCZ typically begins with a prodromal phase, marked by behavioral changes, social withdrawal, and a decline in personal performance, which can persist for several months or even years. This phase eventually culminates in a first psychotic episode, the point at which the disorder is most commonly detected.

SCZ is characterized by the presence of symptoms that generally fall into three main categories: positive, negative, and cognitive. Positive symptoms denote the emergence of new phenomena, such as hallucinations, delusions, disorganized thinking and speech, as well as disorganized or abnormal movements, including catatonia. In contrast, negative symptoms involve the loss of previously present functions, manifesting as apathy, social isolation, neglect of personal hygiene, poor speech, difficulty showing emotions, or mental blockages. Mood alterations may be observed, making it challenging to differentiate SCZ from other disorders. Cognitive symptoms also are pronounced and include problems in attention, concentration, and memory.

The prognosis for SCZ patients is significantly influenced by the duration of untreated illness; thus, early detection and diagnosis are crucial. As in the case of other mental disorders, the diagnostic process for SCZ is primarily clinical, since there are no objective medical tests to confirm its presence. Instead, medical testing is employed to exclude non-mental causes such as epilepsy, tumors, infections, or substance use.

Differentiating SCZ from other mental disorders relies on clinical assessment. The appearance of psychotic and affective symptoms can obscure the distinction between SCZ and other psychiatric conditions such as bipolar disorder, psychotic depression, or schizoaffective disorder (characterized by both affective and psychotic episodes). The duration and characteristics of symptoms help differentiate SCZ from other psychotic disorders, like brief psychotic disorder, schizophreniform disorder, or delusional disorder (Ruiloba 2011; Casher and Bess 2019; Oyebode 2022).

### Major depressive disorder

MDD is unipolar depressive disorder, primarily characterized by a persistent low mood and classified within the affective disorders group. MDD can impact various aspects of a patient's life.

Mood alterations in MDD include a depressed mood, irritability, anhedonia (inability to experience pleasure), alexithymia (inability to express or recognize one's feelings), and anxiety. Cognitive functions may also be affected, demonstrated by diminished ability to think, loss of attention, hopelessness, and even suicidal thoughts, which may result in suicide attempts. Patients often neglect usual tasks, personal hygiene, and social interactions. Additionally, sleep and appetite disturbances are common, and somatic symptoms such as digestive issues, headaches, or nonspecific pain may manifest. Risk factors for MDD include biological factors (genetic predisposition, hormones, brain chemicals), sociodemographic variables (gender, age, marital status, socioeconomic status, employment), psychosocial factors (personality traits, social support, life events, stress), and other health and lifestyle factors (other illnesses, poor nutrition, physical inactivity, medication and substance use).

Currently, the diagnosis of MDD is based entirely on clinical symptoms rather than objective medical tests. The evolution and prognosis of MDD can vary; it may lead to treatment-resistant depression, recurrent depressive disorder, or be a precursor to another medical condition. MDD frequently occurs in patients with other mental disorders or within the context of non-mental diseases (Ruiloba 2011; Casher and Bess 2019; Oyebode 2022).

### Bipolar disorder

BD is also a member of the mood disorder family. Individuals with BD experience significant difficulties in regulating their mood, alternating between depressive episodes and periods of mania or hypomania.

Manic episodes are marked by elevated or irritable mood, grandiosity, increased energy, reduced need for sleep, accelerated thinking and speech, distractibility, and an increase in personal projects. These symptoms can lead to behaviors resulting in substantial financial expenditures or sexual disinhibition. The presence of psychotic symptoms during manic episodes is not uncommon. Sometimes, manic symptoms may coexist with depressive symptoms, resulting in a mixed affective state. Emotional lability, where euphoria alternates with crying, is frequently observed during this phase. The complex etiology of BD is influenced by both genetic vulnerability and various environmental factors.

Diagnosing BD is challenging, as the initial symptoms can be easily misleading to clinicians. It is crucial to rule out organic pathologies, such as neurological illnesses or infections. BD often begins with a depressive episode, which can be mistaken for major depressive disorder. The emergence of psychotic symptoms can also lead to confusion with schizophrenia. Mixed affective states and behavioral alterations may be mistaken for personality disorders or substance use disorders. Therefore, a longitudinal diagnosis, monitoring the patient over time, is often necessary in order to achieve an accurate diagnosis (Ruiloba 2011; Casher and Bess 2019; Oyebode 2022).

### Posttraumatic stress disorder

Patients with PTSD exhibit specific symptoms tied to a traumatic event, such as a violent accident, assault, natural disaster, or combat. PTSD is characterized by intrusion symptoms, such as flashbacks, where the individual involuntarily relives the trauma through vivid, retrospective scenes during the day or in dreams. In some patients, anhedonia, dysphoric moods, and altered social cognition are predominant. Avoidance of situations or objects associated with the trauma can severely restrict the patient’s life. Consequently, the individual remains in a heightened state of alertness, overreacting to loud noises, experiencing insomnia, and irritability. In some cases, PTSD may be accompanied by dissociative symptoms. These symptoms typically manifest within six months of the traumatic event; however, in a minority of cases onset of the disorder can be delayed.

Accurate PTSD diagnosis requires a careful analysis of symptoms and the chronology of events. PTSD often coexists with other psychiatric disorders such as depression, anxiety, or substance use disorder, but also with some somatic comorbidities, complicating its course and prognosis (Casher and Bess 2019; Sadock and Sadock 2015).

### Challenges in diagnosing psychiatric disorders: an evaluation

Psychiatric disorders remain without the support of complementary tests for definitive diagnoses. Specifically, diagnostic processes in psychiatry are purely clinical, lacking objective tests for their confirmation. In the assessment of psychiatric disorders, complementary tests are often performed to rule out non-mental causes of a patient's symptoms. Commonly utilized tests include imaging, blood, urine, and drug analysis. Once physical causes are excluded, the diagnosis is presumed to be psychiatric; however, there are no tests that can definitively confirm it.

Therefore, thorough clinical interviews and accurate mental state examinations (MSE) are essential for effective diagnosis of psychiatric disorders. The MSE provides a snapshot of the patient's mental state at the time of evaluation, assessing various aspects, such as consciousness, attention, memory, attitude, language, perception, mood, and reality judgment. Collecting all symptoms, the chronology of events, family history, and descriptions from close associates is crucial for a patient comprehensive evaluation. Analysis of these variables relies heavily on the clinician's impression and expertise, making the diagnosis inherently subjective. Consequently, different psychiatrics may offer varying opinions on the same clinical case. Additionally, psychiatric disorders can manifest differently across cultures due to varying values and beliefs, which complicate the diagnostic process even further.

To introduce some objectivity, classification systems have been established, although they remain contentious. Currently, diagnostic manuals such as the DSM, by the American Psychiatric Association, and the ICD, by the WHO, are widely used. However, these classification systems do not always agree on various aspects. Each new edition of these classification systems enhances the accuracy of mental health diagnoses. This accuracy can be measured using the kappa coefficient (K), which evaluates the agreement between different professionals'assessments. Higher kappa values indicate increased diagnostic accuracy. However, the diagnostic criteria in the DSM or ICD should not be applied rigidly as checklists leading to unquestionable diagnoses. They should serve as initial guidelines, supplemented by the patient's MSE. Recent classifications have included dimensional diagnostic strategies as alternatives to categorical ones, improving inter-observer reliability and offering greater value for treatment and prognosis. Dimensional diagnosis allows for phenotype classification and, consequently, precision medicine. These dimensions are trans-diagnostic (e.g., emotional regulation, negative affect, executive functions), but enable more precise therapeutic strategies. Nevertheless, categorical models still predominate, even though dimensional approach represent an advancement in the conceptual understanding of pathologies.

Classifications often fail to capture the complexity of psychiatric disorders. Symptoms are highly nonspecific and largely subject to the clinician's interpretation, unlike in other medical specialties. Frequently, clinical observation alone is insufficient for an accurate diagnosis. Consequently, initial diagnoses often evolve based on the disease development. Due to these diagnostic challenges, treatment choices are frequently symptom-based rather than diagnosis-based. Given these complexities, the diagnostic process in is intricate, minimally objective, and generally slow.

Metabolomics has emerged as a promising approach in psychiatry, offering insights into the biochemical alterations associated with mental disorders by analyzing small-molecule metabolites in biological samples (Nedic Erjavec et al. 2018). Blood and blood-derived samples, such as plasma and serum, are particularly valuable due to their minimally invasive collection and their reflection of systemic metabolic changes. Therefore, metabolomic studies can provide insights into the metabolic changes underlying these conditions, and identify specific biomarkers associated with psychiatric disorders, which could lead to more accurate and early diagnosis. Early diagnosis, however, can be crucial for the patient's prognosis. Therefore, there is a critical need for identifying and integrating objective potential biomarkers of psychiatric disorders, that can enhance diagnostic accuracy, identify at-risk individuals before the full onset of symptoms, guide personalized treatment strategies, improve patient outcomes, and minimize drug side effects (Insel 2010; Lener et al. 2017). However, integrating such tools into clinical practice faces significant limitations due to absence of a definitive diagnostic gold standard in psychiatry, which creates a moving target for biomarker validation. The lack of stability of diagnostic criteria influences the identification and application of metabolomic findings into clinical practice.

In addition, before biomarkers can be reliably integrated into clinical practice, they must undergo rigorous validation processes. Validation ensures that biomarkers are accurate, reliable, and clinically useful. Without validation, there is a risk of false positives or false negatives, which can lead to incorrect diagnoses and inappropriate treatment (Micheel et al. 2012). Likewise, validated biomarkers must demonstrate robustness and reliability across diverse populations, including different age groups, ethnicities, and genders (Pepe et al. 2004), as well as meet regulatory standards set by bodies such as the U.S. Food and Drug Administration (FDA) or the European Medicines Agency (EMA). These standards require rigorous validation to demonstrate the safety, efficacy, and reliability of the biomarkers (Woodcock and Woosley 2008). Furthermore, validated biomarkers can contribute to more efficient and cost-effective healthcare, by reducing the need for extensive diagnostic testing, minimize the trial-and-error treatment approach, and decrease the overall burden on healthcare systems (Breen et al. 2016). On the other hand, without validation, biomarkers may lead to misdiagnosis and inappropriate treatment, resulting in increased healthcare costs and poor patient outcomes. Thus, the validation of potential diagnostic biomarkers is essential for ensuring their accuracy, reliability, generalizability, and clinical utility. Without validation, the prospective benefits of biomarkers remain unfulfilled.

## Literature search strategy and experimental design

The objective of this comprehensive review was to explore validated metabolomics research, with a focus on specific psychiatric disorders and the types of validation approaches employed. We limited our analysis to peer-reviewed studies that incorporated independent cohort validation, cross-validation, or external validation. Owing to the limited availability of such studies, the majority of validated research has centered on major depressive disorder and schizophrenia, while bipolar disorder and posttraumatic stress disorder have received comparatively less attention. Biomarkers were considered validated if they demonstrated reproducibility in independent cohorts and showed consistent biological relevance across multiple datasets.

This review analyzed the existing literature on metabolomic validation studies in psychiatric disorders. A comprehensive literature search was conducted using multiple databases (PubMed, Scopus, Google Scholar), to identify all validation studies, with no time constraints up to July 2024. The terms used for the literature search included a combination of keywords, such as “validation”, “metabolomics”, “schizophrenia”, “depression”, “bipolar disorder”, “posttraumatic stress disorder”. The study included only the original scientific papers, written in English, applying a metabolomics approach, either targeted or untargeted analysis (Casher and Bess 2019) (Table 1), using all analytical techniques and biological matrixes (blood and blood derived samples, urine, cells, exosomes) (Table 1).

**Table 1:** Overview of analysis types, biological matrixes, and analytical techniques in metabolomic validation studies

Disorder	Sample type	Analysis	Analytical technique*	References
SCZ	Serum-derived exosomes	Targeted	UPLC-QTRAP-MS/MS	Du et al. 2021)
	Plasma	Untargeted	GC–MS	Karahalil et al. 2022)
	PBMC	Untargeted and targeted	GC-Q-MS	Liu et al. 2014)
	Serum	Targeted	UPLC-QqQ-MS	Qing et al. 2022)
	Serum	Untargeted	UPLC-QOrbitrap-MS	Wang et al. 2019)
	Serum	Untargeted	UHPLC-HRMS	Ye et al. 2024)
MDD	Plasma	Targeted	UPLC-QqQ-MS/MS FIA-MS/MS	Ahmed et al. 2019)
	Plasma	Targeted	LC–MS/MS, FIA-MS/MS, LC-QTRAP-MS	Czysz et al. 2019)
	Serum	Untargeted and targeted	LC-QTOF-MS/MS	Lee et al. 2022)
	Plasma	Untargeted and targeted	UPLC-QTOF-MS	Liu et al. 2015)
	Plasma	Targeted	(HP)LC–MS	Ogawa et al. 2018)
	Plasma	Targeted	GC–MS, LC-QqQ-MS/MS	Pan et al. 2018)
	Plasma	Targeted	LC-QqQ-MS	Setoyama et al. 2016)
	Urine	Untargeted	GC-Q-MS, NMR	Zheng et al. 2013a)
BD	Serum-derived exosomes	Targeted	UPLC-MS/MS	Du et al. 2022)
	Urine	Untargeted	NMR	Zheng et al. 2013b)
PTSD	Plasma	Untargeted	UHPLC-MS/MS^2^, GC–MS	Dean et al. 2020)
	Plasma	Untargeted	HPLC-QTOF-MS, GC-Q-MS	Konjevod et al. 2021)
	Plasma	Untargeted	UPLC-MS/MS, GC–MS	Mellon et al. 2019)
MDD, BD	Urine	Untargeted	GC-Q.MS, NMR	Chen et al. 2015)
	Dried blood spots	Targeted	FIA-MS/MS, LC–MS/MS	Tomasik et al. 2024)
SCZ, MDD, BD	Plasma	Untargeted and targeted	CE-TOF–MS, HPLC-ECD	Kageyama et al. 2017)

*UPLC ultra-performance liquid chromatography, *QTRAP* quadrupole ion trap, *MS* mass spectrometry, *MS/MS* tandem mass spectrometry, *GC* gas chromatography, *Q* quadrupole, *QqQ* triple quadrupole, *UHPLC* ultra-high-performance liquid chromatography, *HRMS* high-resolution mass spectrometry, *FIA* flow injection analysis, *LC* liquid chromatography, *QTOF* quadrupole time-of-flight, *HPLC* high-performance liquid chromatography, *NMR* nuclear magnetic resonance, *CE* capillary electrophoresis, *TOF* time of flight, *ECD* electrochemical detection

Each analytical technique offers distinct strengths and limitations. Liquid chrmomatography (LC) coupled with mass spectrometry (MS) provides broad compound coverage and high sensitivity, though it is prone to ion suppression Gas chromatography (GC) coupled with MS excels in detecting volatile compounds and organic acids but requires derivatization, while flow injection analysis (FIA)-MS supports high-throughput workflows but sacrifices resolution, making it suitable for clinical screening. Capillary electrophoresis (CE) coupled with MS is effective for detecting ionic and small polar metabolites, though its sensitivity is relatively low. Lastly, NMR stands out for its reproducibility and non-destructive nature, albeit with lower sensitivity, making it best for longitudinal studies (Nedic Erjavec et al. 2018; Nanita and Kaldon 2016; Plumb et al. 2023).

Nearly half of the research studies included in this review performed LC–MS based metabolomics on plasma samples (Kupfer and Regier 2011; Casher and Bess 2019). Plasma samples are commonly used in studies of psychiatric disorders due to their easy and non-invasive availability. Of the 22 included validation studies, most of them (Kupfer et al. 2013) used plasma as the primary biological matrix for metabolomic analyses, followed by serum and urine. Depending on the biological question that needs to be resolved, two main metabolomic approaches might be applied: untargeted and/or targeted metabolomic analyses. While untargeted metabolomics is hypothesis-generating, unbiased analysis, targeted analysis is hypothesis-driven analysis of specific, known metabolites (Nedic Erjavec et al. 2018). Both untargeted and targeted metabolomic analyses were equally represented in the studies (40.9% each). However, only 18.2% of the articles employed both metabolomic approaches. Likewise, different analytical techniques might be used in metabolomic studies, including NMR and MS. NMR is a reproducible and quantitative technique but has low sensitivity. In contrast, MS can detect low-concentration metabolites, although its quantitation is more complex than NMR. MS can be coupled with separation techniques like GC, LC, or CE. GC–MS is characterized by straightforward compound identification. However, GC–MS requires volatile compounds, often requiring complex sample preparation, which limits its use to a subset of compounds. LC–MS, with less demanding sample preparation, is suitable for analyzing a broad range of compounds, from semi-polar to non-polar metabolites. Therefore, LC–MS has broader applicability than GC–MS. Although CE-MS is more technically demanding and demonstrates lower sensitivity and higher variability, it is useful for profiling native peptides and secondary metabolites.

Each platform has its own strengths and limitations in terms of sensitivity, resolution, and the classes of metabolites it can reliably detect. As a result, studies employing different technologies may generate non-overlapping metabolite profiles, making it difficult to draw consistent conclusions. Even within the same class of analytical platforms, the lack of standardized protocols for sample preparation, data acquisition, and metabolite identification introduces significant variability (Pinu et al. 2019; Li et al. 2024). Without standardized workflows, even studies using the same analytical platform may report divergent findings, limiting the reliability of cross-study comparisons and the identification of consistent biomarkers (Pinu et al. 2019; Li et al. 2024). Therefore, combining different analytical platforms is recommended in order to expand metabolite coverage in untargeted metabolomics (Nedic Erjavec et al. 2018). Regarding metabolomic validation studies analyzed in this review, 45.5% of them employed more than one analytical platform.

## Validation studies

### Types of validation

**Fig. 2:**
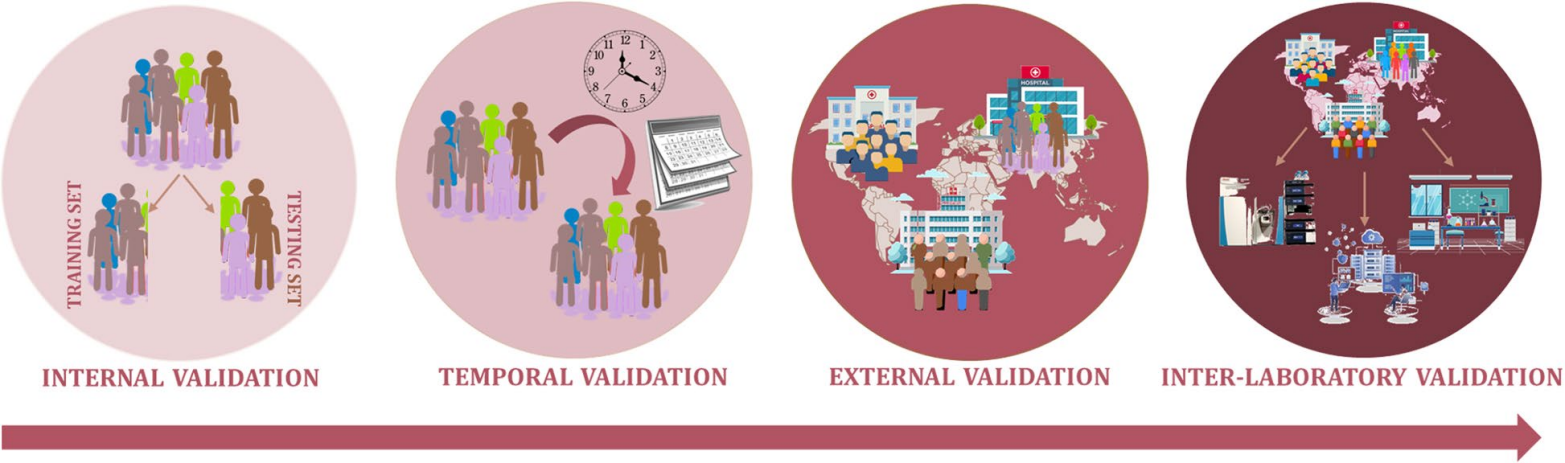
Schematic representation of biomarker validation, arranged sequentially from the least to the most stringent type

The validation of biomarkers for their introduction into the clinical practice can be done internally, over the time (temporal validation), under conditions distinct from those of the initial study by using different set of samples (external validation), or in different laboratories (inter-laboratory validation) (Izumi et al. 2019; Hermans et al. 2016) (Fig. 2).

Typically, internal validation is the most common validation type used in metabolomic research. This approach typically involves two cohorts of samples, a development or training set and a validation or test set, which are analyzed separately. Usually, the participants are randomly assigned to these groups or cohorts and samples are obtained from a single research center, hospital, or laboratory (Cuncic 2024). In this way, more specific metabolites, characteristic for certain population, ethnicity, gender, or even lifestyle are obtained. The internal validation is performed to analyze data without including additional variables (Elias 2024), and provides information about the reproducibility of the analysis (Ramspek et al. 2021). It ensures consistency within a single study, focusing on quality control (QC) samples, technical replicates, method validation, and internal standards. However, internal validation, particularly with small sample sizes, is often insufficient. This limitation arises from the lack of testing in diverse, real-world conditions, where variations in patient demographics and technical factors can influence outcomes. As a result, without additional validation, the findings may capture only study-specific biases.

Temporal validations are often considered in the midway between internal and external validation, because they provide information about reproducibility and generalizability (Hermans et al. 2016; Ramspek et al. 2021). Temporal validation studies the same patients included in the development set, or new patients who are included in the same study, but sampled at different time point(s) (Ramspek et al. 2021).

External validations assess the generalizability and robustness by examining findings under conditions distinct from those of the initial study, on independent sample sets or external cohorts, or comparing obtained findings with other studies. Independent sample sets are referred to testing the models or findings on new sets of samples which are equivalent to the development population, but not used in the original study. On the other hand, for external cohorts the data from different populations or conditions are used in order to ensure that the results are broadly applicable. They usually include patients that come from different countries, different types of care facilities or have different general characteristics (age, gender, smoking status, diet), in order to provide generalized results in various patient populations (Ramspek et al. 2021). The lack of external validation undermines the reliability of proposed biomarkers, because it remains unclear whether they can perform accurately outside the initial study. For clinical translation, it is essential to demonstrate that biomarkers are consistently effective across different populations.

In inter-laboratory validations, the sample set is analyzed by more than one laboratory or research center, potentially applying different techniques and heterogeneous instruments to ensure robustness, reproducibility, and complete generalization of the findings (Mano 2018; Martin et al. 2015). Inter-laboratory validations ensure reproducibility across different laboratories through round-robin tests, proficiency testing, and standardized procedures. This means that different laboratories analyze the same set of samples and compare results, searching for discrepancies that can highlight methodological differences or issues. In addition, different laboratories can be supplied with blind samples to analyze, and their results are then compared against already obtained values. However, inter-laboratory studies often do not address comparisons between different instruments or methods. Very rarely in these type of studies, methodological differences, as well as confounding factors, such as demographics, sample collection and preparation are taken into the account (Lin et al. 2020). Therefore, standardized protocols in these type of metabolomic studies should have been applied. Additionally, inter-laboratory cross-validations encompass comparisons of obtained findings with results from other studies to verify consistency and reproducibility.

External and inter-laboratory validation studies are rarely conducted in metabolomics, which can result in an increased false-positive results rate (Long et al. 2020). Different analytical techniques might be utilized to replicate obtained results (Cuncic 2024). Employing various analytical techniques to replicate the obtained results enhances the trustworthiness of the findings, and utilization of diverse methodologies bolsters confidence in the validity of the results. It is crucial for biomarker discovery to demonstrate that results can be generalized, and that findings remain valid even in the presence of additional variables, not included in the internal validation (Elias 2024).

Not all biomarker-discovery studies need to be validated by all validation types. Depending on the sample size, research topic and biological question that needs to be answered, sometimes internal validation or only geographic external validation may be sufficient (Ramspek et al. 2021).

### Validation studies in psychiatric disorders

**Fig. 3:**
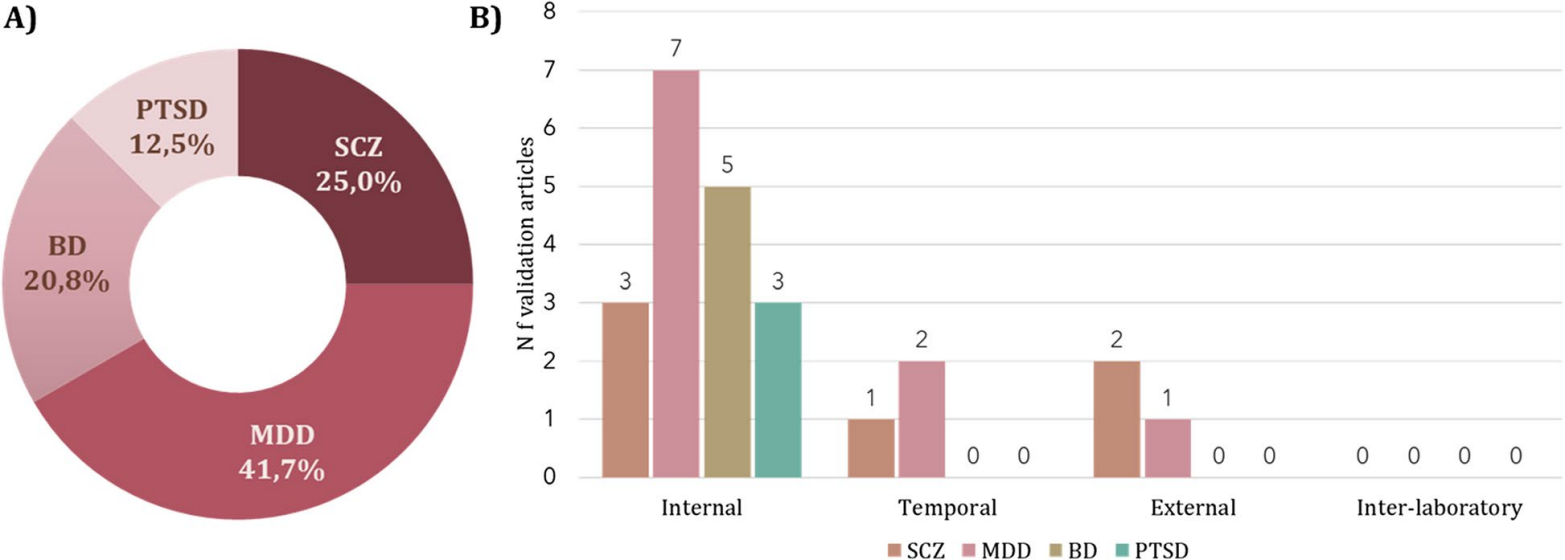
Distribution of validation metabolomic studies according to investigated psychiatric disorder (**A**) and according to the type of validation in each investigated psychiatric disorder (**B**)

**Fig. 4:**
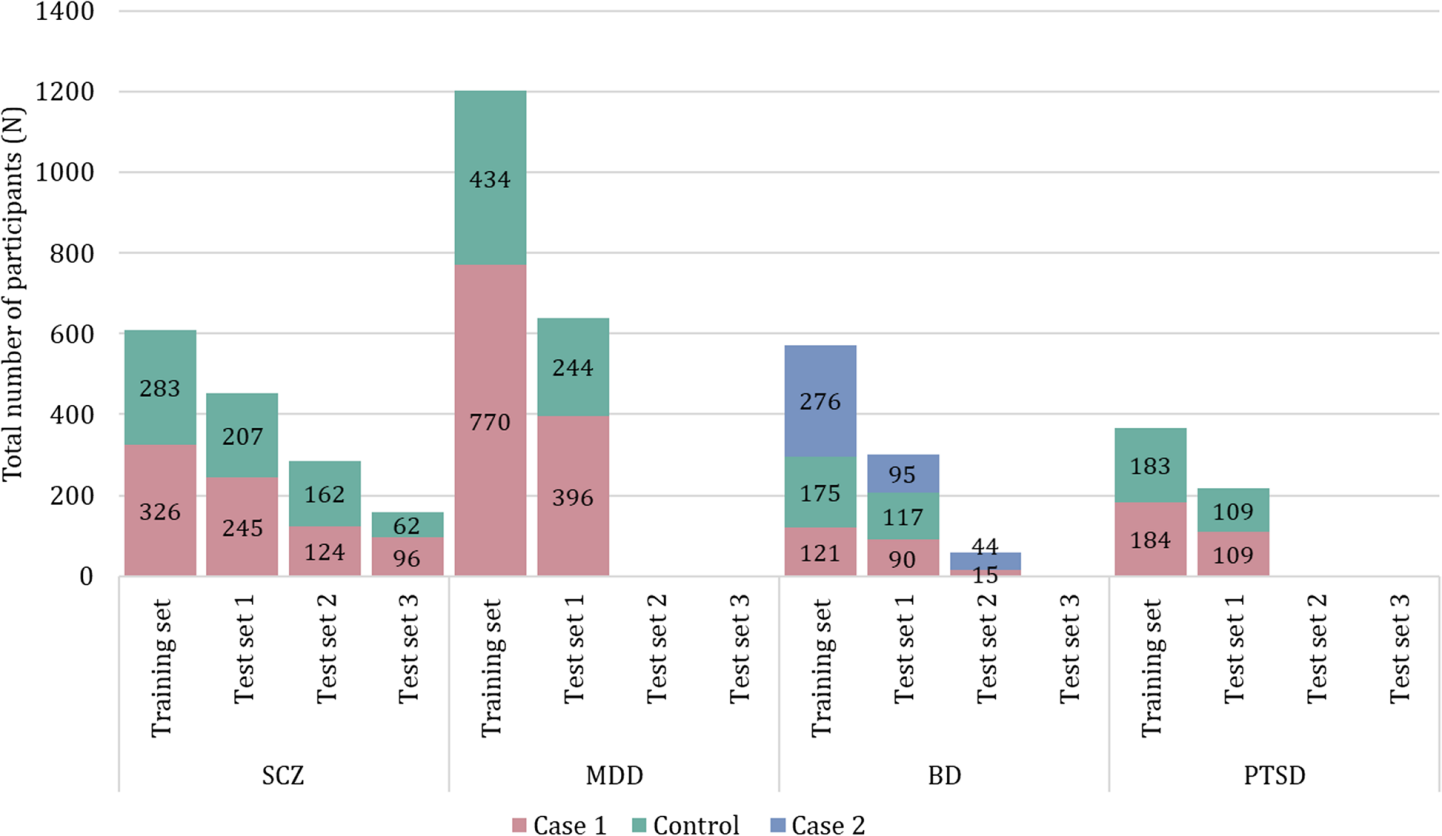
Total number of participants included in reviewed metabolomic validation studies divided per sample set (training or test sets) for each psychiatric disorder. The Case 1 group represents the group of patients with corresponding disorder, the Control group consists of healthy control subjects, while Case 2 group includes individuals with MDD who were studied alongside those with BD

The largest number of validated metabolomic biomarkers in psychiatric disorders have been found for SCZ and MDD, followed by BD and PTSD. PTSD is the least studied of the psychiatric disorders reviewed in the present work (Dean et al. 2020; Konjevod et al. 2021; Mellon et al. 2019) (Figs. 3 and 4). Common metabolites shared between disorders are most pronounced between SCZ and BD, SCZ and MDD, and MDD and BD, reflecting the greater depth of research and diverse validation approaches applied to these conditions compared to PTSD. Furthermore, only a few analyzed articles investigated metabolite changes as a response to specific treatment, performing temporal validation (Karahalil et al. 2022; Ahmed et al. 2019; Czysz et al. 2019), mostly in SCZ and MDD patients.

For most of the psychiatric disorders reviewed in this article, validated metabolomic biomarkers have been identified in only a single study. However, subsequent studies on the same disorders have not replicated their results, highlighting the need for further validation research. These discrepancies in findings across studies may arise from differences in experimental designs, analytical methodologies, and data processing techniques. Similarly, the majority of the validation studies encompassed internal validation with a single training set and a single test set. However, three validation studies deviated from this norm—two belonging to SCZ and one to BD. On average, the training sets comprised the largest number of participants when compared to the other test sets (Fig. 4).

#### Schizophrenia

Due to the complexity and unclear etiology of psychiatric disorders like SCZ, not many metabolomic studies have performed validation. Recently, an external validation metabolomic study, which was published by Du and colleagues (2021), showed blood exosome-derived metabolites that distinguished between SCZ samples and healthy control (HC) subjects recruited from three different centers (Du et al. 2021). The study was comprised of a training set (78 SCZ patients, 66 HC) and three test sets (107, 104, and 96 SCZ patients and 62, 142, and 62 HC, respectively). The biomarker panel, obtained by LC–MS, included 25 exosome-derived metabolites, of which 15 were downregulated and 10 were upregulated. These metabolites are involved in glycerophospholipid metabolism, phenylalanine, tyrosine, and tryptophan biosynthesis, taurine and hypotaurine metabolism, aminoacyl-tRNA biosynthesis, phenylalanine metabolism, arginine metabolism, glycerolipid metabolism, histidine metabolism, ether lipid metabolism, arginine and proline metabolism, as well as primary bile acid biosynthesis (Du et al. 2021). Another external validation study considering different Chinese ethnic groups (30 Miao SCZ and 30 control subjects; 30 Han SCZ and 30 control subjects) collected from two psychiatric hospitals was conducted (Ye et al. 2024). The metabolomic biomarker panel, obtained by LC–MS, included 47 significantly different metabolites in both cohorts. Altered metabolites belonged to the various metabolite classes, including group of fatty acids and derivatives (e.g., indole-3-butyric acid, 2-oxovaleric acid, eicosapentaenoic acid), amino acids (e.g., glutamate, pyroglutamic acid, proline, taurine), and other compound classes (e.g., bilirubin, uric acid, α—tocopherol). Even though some distinct metabolites have been found between these two ethnic groups, external validation showed that there was large number of common metabolites, thus indicating that changes in fatty acid and lipid metabolism are common metabolic changes in the SCZ pathogenesis (Ye et al. 2024).

Moreover, the influence of antipsychotic drugs on the metabolic profile can give an interesting insight into how these drugs might affect or correct altered metabolic pathways in SCZ, identify putative predictive biomarkers, and improve existing treatment approaches to alleviate symptoms characteristic for SCZ patients. In these cases, temporal validation studies are often performed. For example, one metabolomic study (Karahalil et al. 2022) included only female subjects with first-time SCZ, in which plasma samples were collected and analyzed at three different time points: T1: before the treatment with olanzapine, T2: 10 ± 3 days after treatment, and T3: 3 ± 1 months after treatment. It has been shown that 16 metabolites were significantly different between these three time points. The study also evaluated the impact of glutathione transferase mu 1 (*GSTM1)* gene polymorphisms on metabolomic profiles in patients treated with olanzapine, revealing significant differences in metabolite levels. Amino acid tryptophan might represent potential biomarker in metabolome monitoring changes of SCZ patients after olanzapine treatment, in addition to changes in iminodiacetic acid, caprylic acid, urea, L-alanine and phosphoric acids (Karahalil et al. 2022).

Other metabolomic studies in SCZ mostly focused on the internal validation. For instance, internal validation conducted by Qing et al. (2022) included discovery and a validation cohort, in total of 108 SCZ and 108 control subjects (Qing et al. 2022). While the discovery cohort was age and gender matched, in the validation cohort there were significant differences in age and gender due to higher representation of younger females in the control group. Bile acid levels were analyzed using LC–MS targeted metabolomics. Decreased levels of ten bile acids in SCZ patients were found, suggesting gut microbiota alterations (Qing et al. 2022). When their cut-off values have been used in both sample sets, the area under the curve (AUC) scores were above 0.7, suggesting good distinguishing between SCZ patients and control subjects in both cohorts (Qing et al. 2022). Other studies (Liu et al. 2014; Wang et al. 2019) performed internal validation, in which SCZ samples and corresponding HC were recruited from the same hospital and divided into different cohorts. The study by Liu et al. (2014) aimed to identify altered metabolic pathways in the training set (45 SCZ + 50 HC) that would be validated in the test set 1 (24 SCZ + 35 HC) and quantified in the test set 2 (20 SCZ + 20 HC) (Liu et al. 2014), while in the study by Wang et al. (2019), patients and corresponding control subjects were divided on the training set (84 SCZ + 77 HC) and test set, that had smaller number of subjects (35 SCZ + 32 HC) (Wang et al. 2019). Whereas the first study (Liu et al. 2014) performed GC–MS PBMC samples, the study by Wang et al. (2019) (Wang et al. 2019) analyzed serum samples by LC–MS. Liu et al. (2014) found eighteen metabolites in PBMC samples that discriminate between SCZ patients and HC subjects, while decreased levels of three metabolites—pyroglutamic acid, sorbitol, and α—tocopherol —contributed to the highest degree of separation between these two groups in all three cohorts. These metabolites are involved in oxidative stress, neurotransmitter metabolism, and energy metabolism (Liu et al. 2014). In the study by Wang et al. (2019), which was more focused on the serum lipid profile, six different, potential glycerophospholipid biomarkers were found in the serum of SCZ samples and demonstrated good classification in both training and test sets (Wang et al. 2019).

#### Major depressive disorder

Alongside SCZ research, most of the metabolomic validation studies have been conducted on samples obtained from MDD patients. In the study by Liu et al. (2015), internal validation with discovery and validation sets was conducted (Liu et al. 2015). The discovery set included 60 drug-naïve MDD patients and 59 HC, while the validation set enrolled 75 MDD patients and 52 HC patients. Untargeted LC–MS analysis revealed altered metabolites, such as (ether) phospholipids, acyl carnitines, amino acids, free fatty acids, and bile acids. Identified metabolites were next to be validated in different sample batch using targeted metabolomic analysis. Several metabolites, such as glycerophospholipids and acyl carnitines, were validated (Liu et al. 2015). Other study researched altered amino acid profile in subjects with MDD in two independent case–control sample sets (sample set A and sample sat B) (Ogawa et al. 2018). Both, case–control sample sets included large number of samples 147 MDD and 217 HC subjects and 65 MDD and 65 HC subjects, respectively. After LC–MS analysis of plasma samples, two metabolites, glutamate and methionine, were significantly elevated in MDD patients in both sample sets (Ogawa et al. 2018). In the study by Pan et al. (2018), targeted metabolic analysis of 19 plasma metabolites involved in GABAergic, catecholaminergic, and serotonergic neurotransmitter systems on two independent case–control sample sets was performed (50 MDD and 50 HC subjects in the training set, and 49 MDD and 40 HC subjects in the testing set). Interestingly, two analytical methods, GC–MS and LC–MS/MS were used for the determination of metabolic profile (Pan et al. 2018). Four plasma metabolites (γ-aminobutyric acid—GABA, dopamine, tyramine, and kynurenine) have been established as potential plasma biomarkers that could discriminate between MDD patients and control subjects in both the training and testing sets (Pan et al. 2018). In a parallel investigation, Zheng et al. (2013) conducted an internal validation through GC–MS analysis on both training (comprising 82 individuals with MDD and 82 HC subjects) and test sets (including 44 MDD patients and 52 HC subjects) utilizing urine samples. Their analysis identified six pivotal metabolites—azelaic acid, sorbitol, uric acid, quinolinic acid, hippuric acid, and tyrosine—that effectively discriminated between individuals with MDD and controls subjects (Zheng et al. 2013a).

Interestingly, temporal validation of potential MDD metabolomic biomarkers has been performed on plasma samples of a subset of participants from the Combining Medications to Enhance Depression Outcomes (CO-MED) trial population in order to identify predictors or metabolites related to depression recovery (Czysz et al. 2019). The baseline cohort included 159 MDD subjects, while the follow-up cohort, which included 12-week follow-up plasma samples, consisted of 83 MDD subjects. Participants in the CO-MED trial were under medication for one of three pharmacological treatments, including escitalopram monotherapy, bupropion-escitalopram combination, or venlafaxine-mirtazapine combination. It has been reported that higher baseline levels of phosphatidylcholine (PC 38:1) predicted smaller changes in the Quick Inventory of Depressive Symptoms (QIDS), while lysophosphatidylcholine (LPC 20:3) was the most influential individual metabolite, when relative metabolite changes from baseline to study completion were modelled (Czysz et al. 2019).

Two metabolomic studies (Ahmed et al. 2019; Lee et al. 2022) researched altered acylcarnitine levels in MDD. While one study performed internal validation through discovery and validation sets (Lee et al. 2022), the other study performed temporal validation of altered metabolites and researched the effect of citalopram or escitalopram on the plasma metabolic profile of MDD patients (Ahmed et al. 2019). In the first study (Lee et al. 2022), the discovery set was comprised of 32 MDD and 26 control subjects, analyzed by non-targeted metabolic analysis, while the validation set included 44 MDD patients, 35 remission patients and 35 control subjects analyzed by targeted analysis. Acetylcarnitine levels were significantly lower in the MDD group compared to the control and remission groups, suggesting its potential as a diagnostic and predictive biomarker for MDD (Lee et al. 2022). The second study (Ahmed et al. 2019) performed targeted LC–MS analysis in different phenotypes of MDD, which might lead towards a more personalized treatment approach for different subclasses of MDD. However, this study showed that several short-, medium-, and long- chain acylcarnitines were altered in all studied MDD phenotypes at the baseline and after 8-weeks follow-up, suggesting that these metabolites might represent potential biomarkers of MDD.

#### Bipolar disorder

Due to the shared disease mechanisms and clinical symptoms between MDD and BD, several metabolomic studies initially aimed to differentiate between these two disorders. Therefore, three validated metabolomic studies (Setoyama et al. 2016; Chen et al. 2015; Tomasik et al. 2024) have been conducted with the aim of finding potential biomarkers that would help discriminate between MDD and BD and prevent false diagnoses, especially regarding BD, which is often diagnosed as MDD.

A study by Setoyama and colleagues (2016) performed LC–MS external validation, whereas MDD patients and subjects with BD have been recruited from three different centers. Data set 1 included 26 medication-free psychiatric patients; data set 2 consisted of 23 drug-medicated MDD patients; and data set 3 consisted of 27 medicated or not-medicated MDD patients and 14 BD patients (Setoyama et al. 2016). It has been shown that several metabolites were contributing to the Partial least squares (PLS) regression model regarding the Hamilton Rating Scale for Depression and/or Patient Health Questionnaire (PHQ)−9 in all three data sets. These metabolites were 3-hydroxybutyrate, citrate, betaine, creatinine, and GABA (Setoyama et al. 2016). Another metabolomic study (Tomasik et al. 2024) performed internal validation, whereas participants were divided into discovery (174 MDD and 241 BD subjects) and validation cohort that contained considerable much smaller number of samples (21 MDD and 9 BD subjects). The obtained results showed 17 altered metabolites that might represent potential biomarkers, being ceramide d18:0/24:1 the most significant. Other altered metabolites belonged to the group of triglycerides, phosphatidylcholines, sphingolipids, amino acids, bile acids, and other metabolite classes (Tomasik et al. 2024). Furthermore, in the study by Chen and colleagues (2015), NMR analysis of urine samples has yielded 20 altered metabolites, differentially expressed between MDD and BD subjects, while a biomarker panel consisting of six metabolites – propionate, formate, β-alanine, dihydroxybutanoic acid, 2–4-dihydroxypyrimidine and phenylalanine—was validated in the testing cohort with predictive accuracy near 80% (Chen et al. 2015).

Three metabolomic studies performed external (Kageyama et al. 2017) or internal (Du et al. 2022; Zheng et al. 2013b) validation on subjects with BD and control samples (Du et al. 2022; Zheng et al. 2013b; Kageyama et al. 2017). All three studies divided subjects into training (6 BD and 19 HC subjects (Kageyama et al. 2017); 12 BD and 16 HC subjects (Du et al. 2022); 60 BD and 62 HC subjects (Zheng et al. 2013b)) and test sets (16 BD and 11 HC subjects (Kageyama et al. 2017); 20 BD and 24 HC subjects (Du et al. 2022); 26 BD and 34 HC subjects (Zheng et al. 2013b)). Three different matrixes: plasma, serum-derived exosomes, and urine, were analyzed by CE-MS, LC–MS and NMR, respectively. In the metabolomic study by Kageyama et al. (2017) Kageyama et al. 2017), external validation was performed, with the subjects in the two cohorts collected from different hospitals. The authors reported challenges in replicating findings such as altered citrulline levels and the candidate biomarker N-methyl-norsalsolinol in different sample sets (Kageyama et al. 2017). Furthermore, the 15 altered serum exosomal metabolites found by Du et al. (2022) (Du et al. 2022) (chenodeoxycholic acid, lysophosphatidylethanolamines- LPE (18:0) and LPE (14:0), N-acetylmethionine, 13-oxoode, glycine, 1-naphthylacetic acid, 2-aminoethanesulfonic acid, d-2-aminobutyric acid, LPC (18:0), LPC (20:1), biopterin, phosphoric acid, glucosamine, and PAF C-16) might be used as a biomarker panel to differentiate between BD patients and control subjects, showing good predictive AUC score in training (0.838) and testing sets (0.971). Finally, internal validation metabolomic study performed by Zheng et al. (Zheng et al. 2013b) identified four metabolites (α-hydroxybutyrate, choline, isobutyrate, and N-methylnicotinamide) as a potential urinary biomarkers for BD, showing good classification in the training (AUC = 0.89) and testing sets (AUC = 0.86).

#### Posttraumatic stress disorder

Due to the complexity of etiology and pathogenesis, as well as heterogeneous symptomatology of PTSD, metabolomic studies on this psychiatric disorder remain scarce, particularly those incorporating validation measures.

To date, only three metabolomic validation studies on PTSD have been published (Dean et al. 2020; Konjevod et al. 2021; Mellon et al. 2019). All three published studies performed LC–MS and GC–MS analyses on plasma samples of PTSD subjects and corresponding control subjects, divided into 2 or 3 cohorts: the first cohort, which is the discovery or exploratory cohort; the second cohort, which is the recalled cohort (only in the study by Dean et al., 2020); and the third cohort, which is the validation or test cohort. Thus far, internal validation methods have been predominantly employed to confirm potential biomarkers of PTSD. The validation study by Dean et al. (2020) was divided into a discovery and recalled cohort with 83 combat related PTSD patients and 82 combat exposed controls, and validation cohort with 26 PTSD subjects and 26 combat exposed controls (Dean et al. 2020). It was found that the Global Arginine Bioavailability Ratio (GABR), which is defined as arginine/[ornithine + citrulline] and lactate/citrate, was altered in PTSD subjects in all three cohorts (discovery, recalled, and validation); however, while GABR was decreased in PTSD subjects in discovery cohort, it was increased in recalled and validation cohorts. In addition, gamma-glutamyltyrosine was increased in all three cohorts of subjects with PTSD compared to HC subjects. The biomarker panel containing 28 compounds, identified after analysis of the discovery set, showed good performance (AUC = 0,80) in an independent validation cohort (Dean et al. 2020). Another study (Mellon et al. 2019) found altered GABR in subjects with PTSD in both the discovery (51 PTSD and 51 HC subjects) and test cohorts (31 PTSD and 31 HC subjects); however, in the test cohort, that change was not statistically significant. Potential candidate biomarkers that were identified and later validated were pyruvate, lactate, fatty acids, and hypoxanthine. Levels of pyruvate, lactate, and hypoxanthine were significantly increased in patients with PTSD compared with HC subjects, while levels of fatty acids were decreased in plasma samples of PTSD subjects. Other altered metabolites that belong to the group of sphingolipids, carnitines, sterols, amino acids, and nucleotides were not replicated in the second cohort (Mellon et al. 2019). Finally, a study by Konjevod et al. (2021) with around 50 PTSD and 50 control samples in both cohorts, identified statistically significant metabolites that belong to the group of glycerophospholipids, sugars, amino acids, acyl carnitines, phosphosphingolipids, fatty acids, steroidal glycosides, and carboxylic acids (Konjevod et al. 2021). However, only two metabolites after LC–MS/MS analyses have been validated: LPC (18:1) and LPE (18:1), which in both cohorts had increased levels in subjects with PTSD compared to control subjects (Konjevod et al. 2021). Interestingly, sphingosine-1-phosphate was identified, but not validated across PTSD studies (Konjevod et al. 2021; Mellon et al. 2019).

## Common altered metabolic pathways in psychiatric disorders

**Fig. 5:**
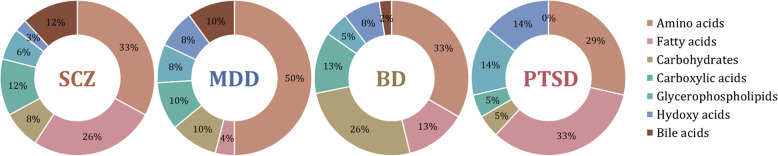
Percentage of the most common validated metabolic classes in the studied psychiatric disorders

**Fig. 6:**
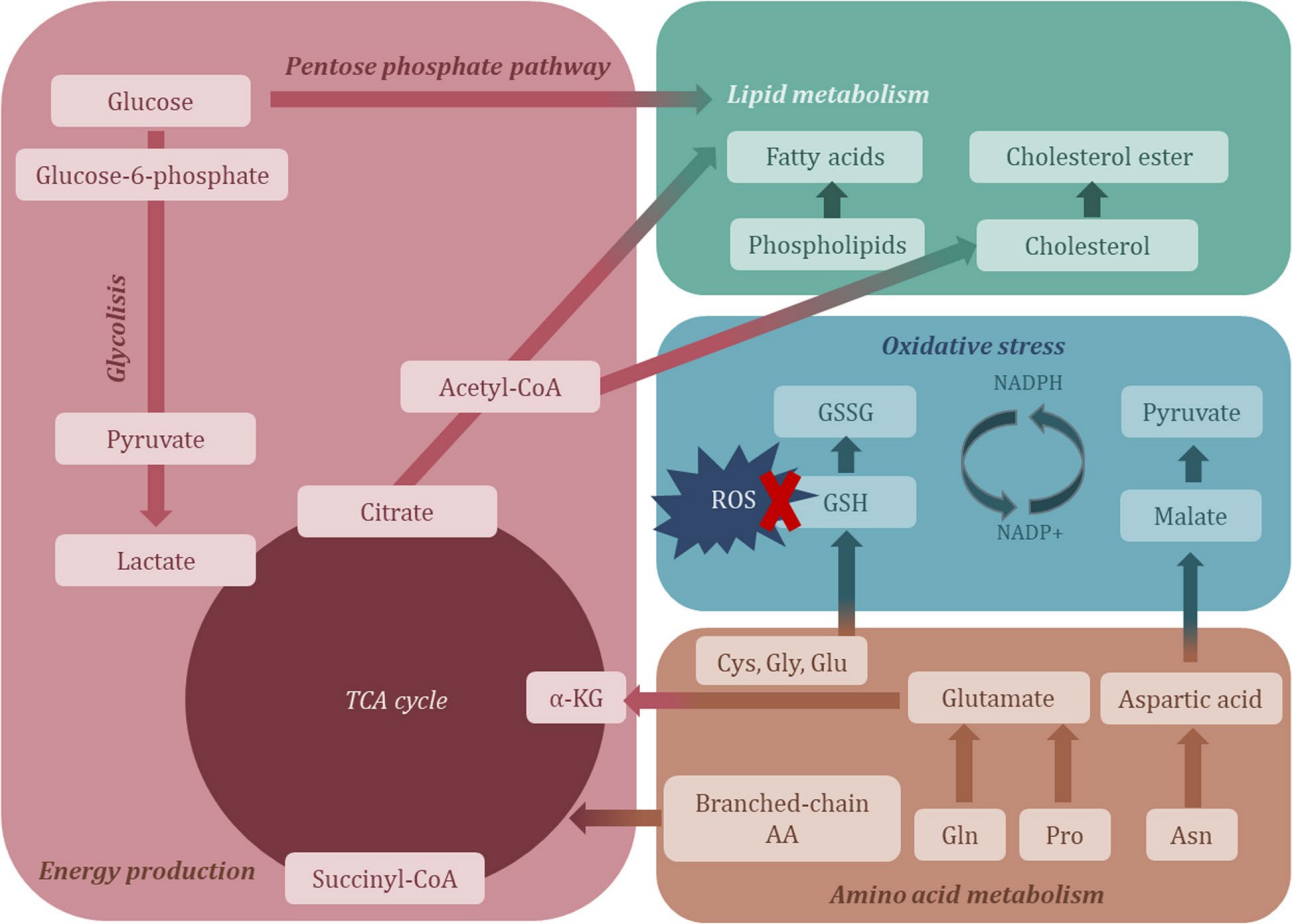
Simplified schematic representation of altered metabolic pathways in psychiatric disorders

**Fig. 7:**
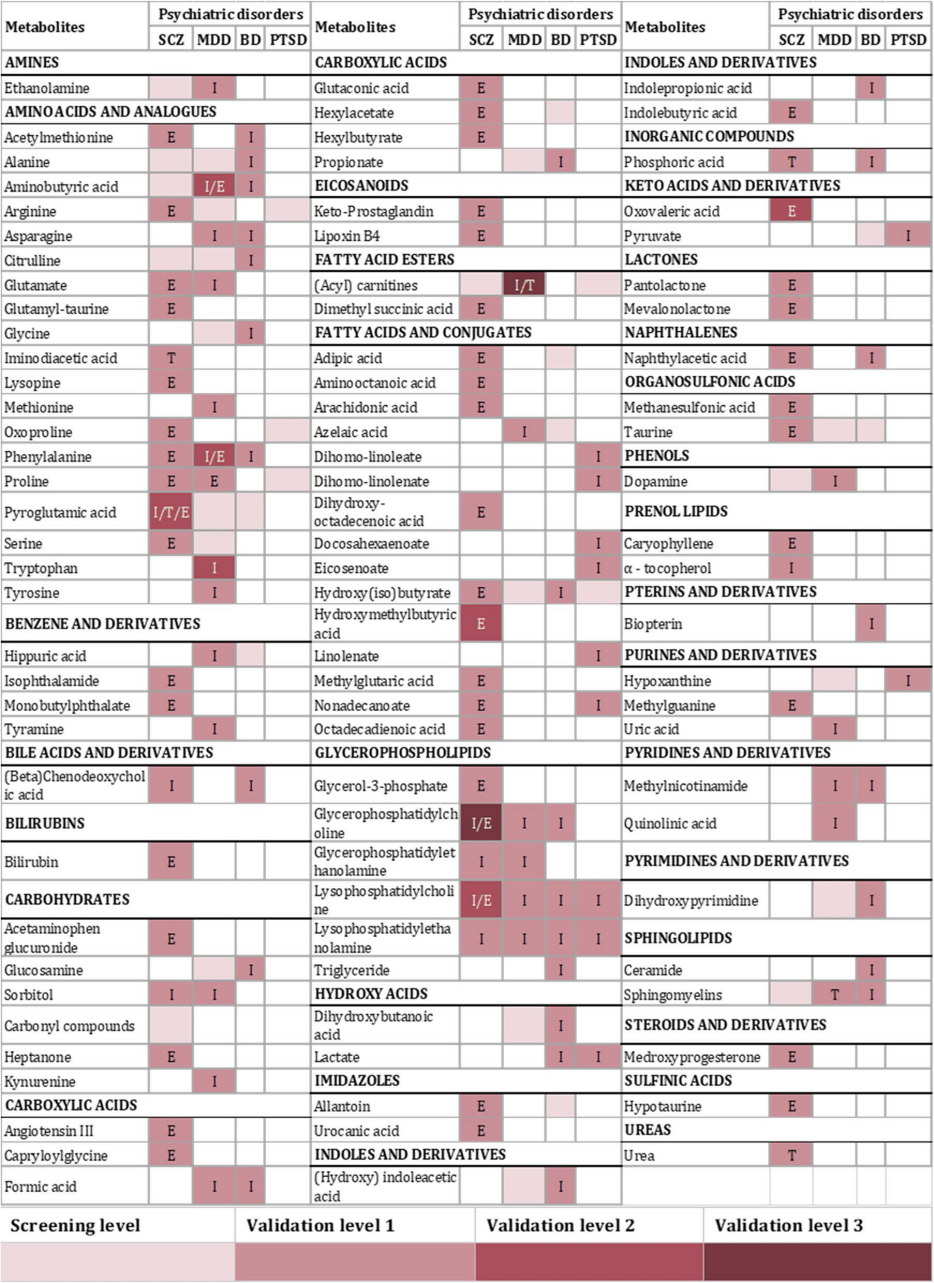
Summarized heat map of analyzed metabolites and their validation level. “I” = internal validation; “T” = temporal validation; “E” = external validation

Numerous metabolic pathways have shown both upregulation and downregulation across diverse psychiatric disorders. Validated metabolomic biomarkers for one, or even all disorders belong to the group of amino acids or their derivatives (Du et al. 2021; Ogawa et al. 2018; Du et al. 2022; Chen et al. 2015), carbohydrates and their conjugates (Liu et al. 2014; Zheng et al. 2013b), as well as to the class of fatty acids, pyridines, sphingolipids and the group of glycerophospholipids (Ye et al. 2024; Liu et al. 2015; Konjevod et al. 2021; Mellon et al. 2019) (Figs. 5 and 7). Reported alterations encompass shifts in lipid metabolism, amino acid metabolism, energy production pathways, carbohydrate metabolism, the kynurenine pathway, oxidative stress pathways, and numerous others (Fig. 6).

In Fig. 7, the summarized heat map illustrates the altered metabolic classes alongside the corresponding identified metabolites, detailing their respective levels of validation. The included metabolites were detected solely at the screening stage without subsequent validation, but also metabolites which were validated through one (Validation level 1), two (Validation level 2), or three independent studies (Validation level 3). The specific type of validation for each metabolite is indicated.

### Lipid metabolism

In various metabolomic studies altered levels of glycerophospholipids and fatty acids have been observed. The biological membranes are composed of phospholipids and their dysfunction and structure modification might represent risk factor for development of certain psychiatric disorder, due to importance of phospholipids in normal brain development and functioning (Du et al. 2021). Glycerophospholipids have an important role in various biological processes, including transportation of the substrates, cell signaling, membrane anchoring, metabolic reactions, apoptosis, and development (Wang et al. 2019; Konjevod et al. 2021). The glycerophospholipids are comprised of four subgroups of metabolites, including PCs phosphatidylethanolamines (PE), phosphatidylserine (PS) and phosphatidylinositol (PI). Among these, PCs, a predominant class in the membranes and circulation, have been particularly scrutinized for their dysregulation, potentially linked to altered synthesis pathways. Concurrently, LPCs derived from PC breakdown or lipoproteins, have also shown perturbed levels in various psychiatric disorders, likely influenced by enzymatic processes such as lecithin-cholesterol acyltransferase (LCAT) and phospholipase A (Wang et al. 2019). However, several studies showed that decreased PC and increased LPC levels might be result of PC degradation due to membrane breakdown and large production of LPC (Wang et al. 2019; Konjevod et al. 2021). This is supported by the proton magnetic resonance spectroscopy (^1^H-MRS), which revealed decreased levels of N-acetyl aspartate, a marker of neuronal integrity (Saccaro et al. 2024). Furthermore, increased production of LPC might affect inflammatory processes and immune system function, (Wang et al. 2019; Konjevod et al. 2021), which is additionally supported because LPC was found to be correlated with white matter integrity in subjects with MDD (Wei et al. 2022). Various neuroimaging studies showed reduction in cortical thickness in subjects with PTSD due to increased proinflammatory cytokine levels (O’Donovan et al. 2015; Miller et al. 2015; Kim et al. 2020), supporting inflammation as an underlying mechanism in PTSD development. Likewise, increased levels of LPCs can induce oxidative stress (Liu et al. 2015), which is pervasive feature across psychiatric disorders including SCZ, MDD and PTSD (Wang et al. 2019; Liu et al. 2015; Konjevod et al. 2021). This is further supported by research indicating increased lipid peroxidation in the prefrontal cortex of individuals with BD (Andreazza et al. 2013) and SCZ (Flatow et al. 2013). Additional findings include associations between frontal lobe phospholipid metabolism and brain structure (Keshavan et al. 1993), as well as reduced antioxidant activity in the prefrontal cortex of SCZ patients (Do et al. 2000).Similarly altered levels of PE and LPE have been reported in aforementioned psychiatric disorders. PEs are the second most abundant glycerophospholipids in the membrane and have been implicated in membrane and mitochondrial integrity, reflecting modifications in phospholipase A2 (PLA2) activity (Konjevod et al. 2021). Hyper-reactivity of PLA2 enzyme lead to degradation of PE and increased production of LPE, which is associated with increased inflammation (Wang et al. 2019; Konjevod et al. 2021; Konjevod et al. 2022). These alterations extend to other glycerophospholipids, such as PIs and PSs, which also play pivotal roles in cellular functions, particularly in inflammatory pathways that are consistently disrupted in psychiatric disorders (Konjevod et al. 2021). In addition, alterations in sphingomyelin (SM) metabolism have been reported in psychiatric and neurodegenerative disorders. SMs are one of the main components of the myelin membranes, thus decreased levels of SMs might indicate decreased myelination. For example, increased lipid peroxidation of the myelin in the prefrontal cortex has been found in subjects with BD (Andreazza et al. 2013). Altered levels of choline, main component of PCs is shown to be altered as well in PTSD, BD and MDD. Phosphatidylcholines represent choline donor to SM, which might affect levels of choline, PC and SM (Wang et al. 2019; Konjevod et al. 2021), while changes in choline levels might be associated with alterations in acetylcholine neurotransmission as well (Zheng et al. 2013b). Likewise, SM might act as LCAT inhibitor and therefore influence PC and LPC levels on the periphery (Wang et al. 2019).

Alongside these lipid alterations, changes in the fatty acid profiles, encompassing both pro-inflammatory saturated fatty acids and anti-inflammatory unsaturated fatty acids, further underscore the multifaceted role of lipid metabolism in psychiatric pathology. Unsaturated fatty acids, derived from the diet are involved in processes such as thermoregulation, energy metabolism, growth and differentiation, intermediary metabolism, thus reduction in levels of unsaturated fatty acids or elevation in levels of saturated fatty acids might cause enhanced inflammation (Mellon et al. 2019). Additionally, elevated lipid peroxidation resulting from the oxidation of polyunsaturated fatty acids has been observed in individuals with PTSD, underscoring the significant role of oxidative stress in the disorder’s pathophysiology (Miller et al. 2018). Changed levels of various fatty acids, including arachidonic, docosahexaenoic acids or their derivatives have been reported on the periphery of various disorders, including SCZ, BD, PTSD, MDD, as well many others like autism, attention deficit hyperactivity disorder (ADHD) and anxiety (Du et al. 2022; Konjevod et al. 2021; Mellon et al. 2019). Phospholipid degradation can disrupt mitochondrial function and impair membrane components vital for energy production. Oxidative stress may increase lipid peroxidation and free radical formation, contributing to altered lipid levels observed in psychiatric disorders. Additionally, lower levels of unsaturated fatty acids may negatively impact dopamine and serotonin signaling, both of which are often disrupted in these conditions (Du et al. 2021; Konjevod et al. 2021; Mellon et al. 2019).

Moreover, acylcarnitines exhibit variable levels in different psychiatric disorders, implicating their role in cellular energy production and cholinergic neurotransmission (Lee et al. 2022; Liu et al. 2015). Altered levels of carnitines have been found in subjects with MDD, PTSD and SCZ (Ye et al. 2024; Lee et al. 2022; Liu et al. 2015; Konjevod et al. 2021; Mellon et al. 2019). Levels of acylcarnitines were reduced in subjects with depression and PTSD (Lee et al. 2022; Liu et al. 2015; Konjevod et al. 2021). However, other studies found increased levels of carnitines in SCZ and PTSD patients compared to control subjects (Ye et al. 2024; Mellon et al. 2019). Another group of metabolites involved in lipid metabolism are bile acids (Qing et al. 2022; Liu et al. 2015). Bile acids are involved in several metabolic processes, including lipid metabolism, glucose and energy metabolism, inflammation, signaling and gene transcription (Qing et al. 2022). Altered bile acid levels have been observed in various psychiatric disorders, yet only one metabolomic study has validated these findings specifically in SCZ serum samples. This study internally validated five bile acids across two cohorts, revealing significantly decreased concentrations of chenodeoxycholic acid, ursodeoxycholic acid, 3β-chenodeoxycholic acid, 7-ketolithocholic acid, and 3-dehydrocholic acid, including total and unconjugated bile acids, in subjects with SCZ compared to HC subjects. It is already well-known that bile acids can traverse the blood–brain barrier, interacting with brain receptors and potentially transmitting critical peripheral information to the CNS. Moreover, chenodeoxycholic acid is an antagonist of N-methyl-D-aspartate (NMDA) and GABA receptors, and therefore reduction in the levels of chenodeoxycholic acid indicate alterations in GABAergic and glutamatergic neurotransmission. Furthermore, gut microbiota is involved in transforming, conjugation and deconjugation of bile acids, in order to produce unconjugated bile acids, followed by processes that produce secondary bile acids (Qing et al. 2022). Thus, alterations in bile acids might be associated with changes in gut microbiota. For example, it has been reported that bile acid 3-dehydrocholic acid was positively correlated with the gut species *Lactobacillus gasseri.* Lower bile acid deconjugation by gut microbiota has been correlated with SCZ relapse. Associations between bile acids and gut microbiota suggest that alterations in gut microbiota, including their gut composition, as well their deconjugative capacity represent potential risk for SCZ (Qing et al. 2022).

Although it is important to point out that even the association between lipid metabolism and psychiatric disorders has been widely reported, many studies fail to adequately account for potential confounding factors such as diet, physical activity, medication and other lifestyle variables. These influences can significantly alter lipid profiles, making it difficult to determine whether observed changes are truly related to the psychiatric condition or simply reflect external factors. Furthermore, there is a notable inconsistency in metabolite changes across different studies, with some reporting opposing trends for the same metabolites. This lack of reproducibility raises concerns about the reliability of these findings and their suitability as diagnostic biomarkers.

### Amino acid and neurotransmitter metabolism

Amino acids constitute a group of metabolites predominantly recognized for their role as fundamental building blocks of proteins; however, they also play diverse essential roles in maintaining homeostasis and normal metabolic function. Beyond their structural function, amino acids serve as essential cell signaling molecules, precursors for numerous hormones and neurotransmitters, and participate in processes such as gene regulation and protein phosphorylation (Wu 2009). Dysregulation of several amino acids have been found in psychiatric diseases discussed in previous sections. Specifically, alterations in phenylalanine, tyrosine, tryptophan, and histidine—amino acids involved in dopamine synthesis—have been implicated in the pathogenesis of SCZ, contributing to hypodopaminergic state (Karahalil et al. 2022; He et al. 2012). Changes in these amino acids could potentially impact dopamine synthesis pathways crucial to SCZ pathophysiology (Karahalil et al. 2022). Furthermore, hippuric acid, a derivative of phenylalanine, has also shown decreased levels in individuals with MDD, suggesting potential inhibition of the entire tyrosine-phenylalanine pathway (Zheng et al. 2013a). Tryptophan, another pivotal amino acid, is associated with serotonin neurotransmitter alterations, and decreased tryptophan levels directly correlate with CNS serotonin dysfunction. Interestingly, studies have reported increased tryptophan levels following olanzapine therapy, which may affect CNS serotonin levels. Tryptophan is part of tryptophan-kynurenine pathway, whereas tryptophan, kynurenine, and product of a kynurenine pathway—quinolinic acid, showed altered regulation in psychiatric disorders. For instance, reduction of tryptophan levels and elevation of kynurenine/tryptophan ratio were observed in subjects with SCZ and MDD compared to control subjects (Karahalil et al. 2022; Liu et al. 2015; Zheng et al. 2013a). For instance, increased levels of L-kynurenine have been associated with alterations in white matter integrity, pointing to a potential connection between inflammation-related metabolic pathways and structural brain changes in MDD (Wei et al. 2022). Similarly, quinolinic acid, was decreased in subjects with MDD, indicating elevation of kynurenine levels in CNS and perturbation of kynurenine metabolism (Zheng et al. 2013a). The altered tryptophan metabolite, indole-3-butyric acid has been associated with gut microbiota, implying that alterations in gut microbiome are implicated in pathogenesis of psychiatric disorder (Ye et al. 2024). In addition, alterations in tryptophan-nicotinic acid metabolism have been implicated, since methylnicotinamide, a product of nicotinamide metabolism is altered in BD, potentially leading to elevated activity of kynurenine pathway (Zheng et al. 2013b).

Moreover, altered levels of amino acids, arginine, alanine, cysteine, glutamine, glutamate, methionine, lysine, proline, serine, valine, and glutamic acid have been reported in several psychiatric disorders. For example, glutamine as a non-essential amino acid, has been singled out as a potential biomarker of BD, since alterations of glutamine and its derivatives have been found in BD post-mortem brain and plasma samples, in comparison to other disorders and control subjects (Chen et al. 2015; Sussulini et al. 2009; Lan et al. 2009). Similarly, proton magnetic resonance spectroscopy showed that proinflammatory cytokines can predict concentrations of brain metabolites such as glutamate, myo-inositol, and N-acetylaspartate in the anterior cingulate cortex of BD patients, indicating a relationship between inflammation and brain metabolism (Poletti et al. 2020).

Altered levels of arginine, its derivative—citruline and lysine have been found to be associated to the dysregulation of nitric oxide metabolism (Du et al. 2021; Karahalil et al. 2022; Kageyama et al. 2017), and GABAergic neurotransmission (Du et al. 2021), while decreased levels of valine might affect serotonin and catecholamine releasement, leading to increased monoamine neurotransmission, which has been associated with BD (Zheng et al. 2013b). In addition, amino acid methionine is susceptible to oxidation leading to production of two diastereoisomers of methionine sulfoxide, catalyzed by methionine-S-sulfoxide reductase enzymes. These enzymes are coded with genes associated with SCZ, its development, and other behavioral phenotypes (Ye et al. 2024). Amino acid proline is an important amino acid that take part in various cellular and metabolic processes, including arginine and glutamate synthesis, immune system, homeostasis and antioxidative processes (Konjevod et al. 2021). Altered levels of proline and its derivative hydroxyproline indicate alterations in proline cycle, proline-glutamate metabolism, glutamatergic signaling and activity of an enzyme prolidase exopeptidase, which have been implied in pathogenesis of various neurodegenerative and psychiatric disorders, including PTSD, MDD, SCZ, and autism (Konjevod et al. 2021).

### Energy metabolism

Metabolites that take part in sugar metabolism, glycolysis, and tricarboxylic acid cycle (TCA) cycle were changed in various sample matrixes in all above-mentioned psychiatric disorders. For instance, studies indicate that peripheral glucose metabolism is disrupted in individuals with BD, PTSD, MDD and SCZ, compared to control subjects. The levels of various metabolites, including octanoic acid, fumaric acid, maltose, valine, inositol, erythrol, gluconic acid, and especially sorbitol, have been changed in various psychiatric disorders, as precursors and by-products of glucose metabolism, assumingly altered as a result of increased glycolysis rate and TCA cycle flux (Liu et al. 2014; Zheng et al. 2013a; Zhang et al. 2016). Likewise, increased levels of octanoic acid and lipoic acid indicate increased activity of glucose metabolic enzymes and stimulation of energy metabolism, which can be seen through higher adenosine triphosphate (ATP) levels and increased expression of enzymes involved in glycolysis and TCA cycle (Liu et al. 2014). Increased levels of ATP lead to production of phosphocreatine, which represent energy reserve, while alterations in levels of fumaric acid influence proline, tyrosine, and aspartate metabolism (Liu et al. 2014), amino acids that are altered in psychiatric disorders. Moreover, increased fumarate levels are likely attributable to elevated succinate levels and an accelerated conversion rate of succinate to fumarate in subjects with MDD, reflecting heightened succinate dehydrogenase activity (Chen et al. 2015). Additionally, elevated lactate and pyruvate levels signify intensified anaerobic glycolysis; however, studies have reported decreased lactate, pyruvate, and acetate levels in individuals with MDD (Chen et al. 2015). It is assumed that lactate affects lipid and glucose metabolism by inhibiting utilization of glucose and long-chain fatty acids for energy metabolism, leading to insulin resistance, increased adiposity, and body mass index in subjects with PTSD (Mellon et al. 2019), or BD and MDD (Chen et al. 2015). Notably, lactate exerts significant influence within the CNS, playing crucial roles in signaling, neuronal activity, cerebral energy metabolism, neuronal gene expression, glucocorticoid sensitivity, and synaptic plasticity. Consequently, fluctuations in lactate levels may lead to alterations in glutamatergic and GABAergic neurotransmission by binding to NMDA and GPR81 receptors (Mellon et al. 2019).

### Oxidative stress

Oxidative stress has been identified as one of the main underlying biological processes in the pathogenesis of psychiatric disorders. It is often characterized by production of reactive oxygen species (ROS) due to inflammation, leading to neuronal and mitochondrial dysfunction (Nedic Erjavec et al. 2018). For instance, post-mortem study revealed heightened oxidative and nitrosative damage to mitochondrial and synaptosomal proteins, along with increased lipid peroxidation in the prefrontal cortex of individuals BD, suggesting that oxidative stress may contribute to subcellular membrane dysfunction in the disorder (Andreazza et al. 2013). Metabolomic studies have unveiled a broad spectrum of altered metabolites associated with oxidative stress. Among these, uric acid and azelaic acid play significant roles in antioxidant defence mechanisms (Zheng et al. 2013a). Uric acid is an end-product of purine metabolism, while azelaic acid is involved in ROS inhibition. They are commonly produced in the environment of an increased oxidative stress. Both of these metabolites were found increased in patients with MDD, indicating higher oxidative stress in these subjects (Zheng et al. 2013a). In addition, in SCZ subjects several altered metabolites were associated with oxidative stress (Liu et al. 2014; Ye et al. 2024), mostly with the lack of antioxidant activity (Liu et al. 2014). For example, increased levels of hydroxylamine and oxidized lipids in SCZ samples indicate increased ROS production and lipid peroxidation (Liu et al. 2014; Ye et al. 2024), while decreased levels of pyroglutamic acid in SCZ and increased levels of α-hydroxybutyrate in BD, reflect potentially lower activity of glutathione, metabolite involved in ROS elimination (Liu et al. 2014; Zheng et al. 2013b). This is supported by increased 4-hydroxynonenal levels – product of lipid peroxidation found increased by 59% in BD patients (Wang et al. 2009). Following the trend, elevated indicators of oxidative stress, such as elevated 4-hydroxynonenal levels and reduced levels of antioxidant metabolites, vitamin E (α—tocopherol and γ—tocopherol) have been found in subjects with SCZ, reinforcing the involvement of oxidative damage in the disorder’s underlying pathophysiology (Liu et al. 2014; Flatow et al. 2013).

## Novel tools for the diagnosis of psychiatric disorders

Traditionally, the diagnosis of psychiatric disorders has been grounded in clinical evaluations, comprehensive interviews, and standardized symptom checklists. However, the artificial intelligence (AI) technologies are being increasingly developed to improve diagnostic precision, forecast patient outcomes, and design individualized treatment plans (Alhuwaydi 2024).

In general, metabolomic studies generates vast and complex datasets consisted of hundreds to thousands of metabolites (Chi et al. 2024), thus, AI might play a significant role in big data analysis. AI in metabolomics can be used in various aspects of metabolomic analysis, and significantly improve the efficiency and accuracy of metabolomic studies, including analytical detection, feature selection and data extraction and pre-processing, biomarker discovery, interpretation of biochemical pathways, as well as integration with other multi-omics data and predictive modelling for personalized approach (Chi et al. 2024; Barberis et al. 2022). The application of machine learning (ML) techniques in metabolomics has grown significantly, particularly for disease diagnosis and biomarker identification. The most common algorithms used are random forest (RF), support vector machine (SVM), artificial neural networks (ANNs), k-nearest neighbors (KNN), and other supervised methods (Barberis et al. 2022; Petrick and Shomron 2022). ML techniques can help with denoising data, correcting batch effects, and identify relevant metabolites. The AI models and algorithms are highly efficient utilizing complex spectral data through improved quantitation, in recognizing specific patterns, characteristic for a certain psychiatric disorder and identifying potential metabolomic biomarkers. For instance, AI methods (e.g., Convolutional Neural Networks) have proven effective in processing, among others, chromatographic peaks, peak deconvolution, and peak selection, while ML methods improve metabolite identification using retention time and structural data. Moreover, in statistical modeling, AI helps to enlighten complex metabolic relationships and identify biomarkers (Chi et al. 2024).

One of the most important goals of metabolomic studies is understanding biological significance of obtained results, which might be enhanced with AI models predicting and reconstructing metabolic pathways involved in the onset and progression of psychiatric disorders (Chi et al. 2024). In addition, AI can analyze and interpret complex datasets from different sources and thus assist in integration with other omics data, such as genomics, transcriptomics, and proteomics to overcome challenges such as varying data scales, complexity, and pathway interactions (Chi et al. 2024). Multi-omics integration methods include sequential and simultaneous approaches, with AI models identifying links between genes, enzymes, and metabolites, leading to novel insights of the disease mechanisms, personalized medicine and drug discovery (Chi et al. 2024). Furthermore, ML has demonstrated considerable promise in psychiatric research, notably in the classification of disorders through magnetic resonance imaging, analyzing expansive datasets, including neuroimaging scans, genetic information, and electronic health records. For instance, ML algorithms have successfully differentiated between brain scans of individuals with SCZ and healthy controls by detecting subtle variations in the brain structure and function (Zhang et al. 2023). Furthermore, ML has been extensively applied to the diagnosis of PTSD, demonstrating efficacy in identifying relevant biomarkers and symptom patterns (Wang et al. 2024; Malgaroli and Schultebraucks 2020). In addition ML models are being developed to anticipate the onset of psychiatric disorders, such as BD and MDD (Yasin et al. 2023; Azevedo Cardoso et al. 2024). Such predictive capabilities facilitate early identification and intervention, potentially mitigating the progression and severity of these conditions.

Natural language processing techniques, another facet of AI, are employed to analyze speech patterns, linguistic usage, and textual data derived from patients, including sources like social media posts and therapy transcripts. These analyses can uncover linguistic markers indicative of psychiatric disorders, such as disorganized speech patterns in SCZ (Deneault et al. 2024) or prevalent negative emotional content in MDD (Han et al. 2022). AI-powered applications are also capable of monitoring and analyzing patients'language use in real-time, thereby assisting in symptom tracking and predicting mood fluctuations or relapse episodes in disorders like BD (Ryan et al. 2020; Saccaro et al. 2021). As application of AI technologies within mental healthcare continue to evolve, they will undoubtedly increase the discovery of new biomarkers, enlighten metabolic pathways involve in the development of psychiatric disorders, and enable more precise disease diagnosis and define better treatment approaches (Alowais et al. 2023).

### Limitations of AI in psychiatric research

Despite the promising potential of AI in psychiatric research, challenges such as data heterogeneity, ethical considerations, overfitting, interpretability and the necessity for more extensive, robust studies persist and must be addressed to fully integrate AI into clinical practice (Barberis et al. 2022; Petrick and Shomron 2022). For example, one of the most pressing technical challenges is overfitting, where models perform well on training data but fail to generalize to new, unseen data. This is particularly problematic in psychiatric applications, where data heterogeneity and relatively small sample sizes can lead to misleading conclusions.

Likewise, AI-driven diagnostics raise concerns regarding privacy, consent, and potential biases in algorithmic decision-making. Patient data, particularly sensitive mental health information must be handled with the utmost confidentiality (Floridi et al. 2018). Furthermore, achieving reliable and generalizable results with AI tools in healthcare requires large datasets from thousands of participants, but most current research is limited to small, single-site studies. This lack of large-scale, multi-center validation limits the clinical usefulness of findings. To overcome this, we need broad collaborations across institutions, diverse and extensive datasets, and standardized protocols for collecting and evaluating data and AI models (Tanaka et al. 2024).

Therefore, until these challenges are systematically addressed, the routine clinical application of AI in psychiatry will remain limited. Continued investment in infrastructure, interdisciplinary collaboration, ethical governance, and rigorous validation studies is imperative to ensure that AI reaches its full potential in clinical practice.

## Conclusion

Diagnosis of psychiatric disorders represent a multifaceted process due to their polygenic nature, heterogeneity, and complex genetic and environmental interactions involved in their development and progression.

One of the key limitations of the selected studies lies in the inherent complexity of psychiatric disorders, which are characterized by unknown etiologies and considerable heterogeneity. Furthermore, the variation in experimental designs, analytical platforms, and inclusion/exclusion criteria across the studies that validated different metabolites significantly hampers the ability to identify and extract any single metabolite as characteristic or specific to a given disorder. This methodological inconsistency presents a major challenge in the search for reliable biomarkers with potential clinical utility. Therefore, while metabolomic research offers valuable insights into altered biochemical pathways, its translation to clinical practice remains limited in due to influence of confounding variables, the absence of standardized methodologies and robust validation processes. Moreover, for a metabolite to serve as a clinically useful biomarker, it must demonstrate both sensitivity and specificity for a particular disorder. This means it should reliably distinguish individuals with the condition from those without, regardless of unrelated variables. However, many proposed biomarkers in psychiatric metabolomics appear to be heavily influenced by non-disease factors such as diet, stress, or medication use. Such variability undermines their diagnostic value, as a true biomarker should reflect the underlying pathology of the disorder rather than general lifestyle or environmental conditions. As a result, rather than proposing specific metabolites as biomarkers, the current body of work primarily contributes to elucidating altered metabolic pathways. Identifying common metabolites across these studies remains difficult. As it is shown previously (Fig. 7), some metabolites appear to overlap across disorders, while others are uniquely reported in individual conditions. However, this uniqueness may reflect a lack of cross-validation rather than true disorder specificity. Therefore, no metabolite was extracted or defined as a biomarker in this review, in order to maintain objectivity and avoid overinterpretation of preliminary or non-replicated findings.

The intrinsic complexity of psychiatric diseases highlights the need for complementary diagnostic tools, such as metabolomics, to assist in the current methodologies. Identifying and integrating potential objective metabolomic biomarkers can enhance diagnostic accuracy, identify at-risk individuals, as well as guide future treatment strategies, in terms of improvement of therapy outcomes and minimization of drug adverse effects. However, it should be evaluated and validated how well such biomarker complements existing diagnostic tools and differentiates between psychiatric disorders that have similar clinical symptoms, such as SCZ, MDD, BD, and PTSD. Nevertheless, our review demonstrated only small number of validated metabolomic studies in psychiatric disorders and emphasized the critical demand for more rigorous research in order to facilitate the introduction of metabolomic biomarkers into psychiatric clinical practice.

## Data Availability

No datasets were generated or analysed during the current study.
